# Human papillomavirus in amniotic fluid

**DOI:** 10.1186/1471-2393-6-28

**Published:** 2006-09-04

**Authors:** Mack T Ruffin, Joanne M Bailey, Diane Roulston, Daisy R Lee, Ruth Ann Tucker, David C Swan, Elizabeth R Unger

**Affiliations:** 1 Department of Family Medicine, University of Michigan, 1018 Fuller St., Ann Arbor, MI 48109-0708, USA; 2 Department of Obstetrics and Gynecology, University of Michigan, Nurse Midwives, F4835 Mott 0264, Ann Arbor, MI 48109-0264, USA; 3 Department of Pathology, University of Michigan, 1301 Catherine Road, Ann Arbor, MI 48109-0602, USA; 4 Division of Viral and Rickettsial Diseases, Centers for Disease Control and Prevention, 1600 Clifton Rd. NE B6109, Atlanta, GA 30329, USA

## Abstract

**Background:**

There is evidence to suggest that human papillomavirus (HPV) can cross the placenta resulting in in-utero transmission. The goal of this study was to determine if HPV can be detected in amniotic fluid from women with intact amniotic membranes.

**Methods:**

Residual amniotic fluid and cultured cell pellets from amniocentesis performed for prenatal diagnosis were used. PGMY09/11 L1 consensus primers and GP5+/GP6+ primers were used in a nested polymerase chain reaction assay for HPV.

**Results:**

There were 146 paired samples from 142 women representing 139 singleton pregnancies, 2 twin pregnancies, and 1 triplet pregnancy. The women were 78% Caucasian, 5% African American, 14% Asian, and 2% Hispanic. The average age was 35.2 years with a range of 23–55 years. All samples were β-globin positive. HPV was not detected in any of the paired samples.

**Conclusion:**

Given the age range, race, and ethnicity of the study population, one would anticipate some evidence of HPV if it could easily cross the placenta, but there was none.

## Background

Infection with human papillomaviruses (HPV) is causal in the development of genital and oral cancers [1,2]. HPV infection is considered a sexually transmitted infection among adolescents and adults [3]. Detection rates of HPV infection by polymerase chain reaction (PCR) have ranged widely between 1% and 20% in newborns of pregnant women without apparent infection in their cervix [4-7] and between 5% and 72% in women with HPV-related cervical diseases diagnosed during pregnancy [8]. Many investigators believe that infants are exposed to HPV and infected during vaginal delivery [9,10]. In contrast, some investigators have suggested that vertical transmission is possible without evidence of vaginal or cervical secretion contact of the fetus [11-16]. However, the number of women studied to confirm placental transmission of HPV has been limited.

The goal of this study was to determine if HPV could be detected in amniotic fluid from women with intact membranes.

## Methods

Human subject approval from the University of Michigan was obtained to use residual amniocentesis samples that were to be discarded. To be eligible for the study, pregnant women had to be undergoing clinically indicated amniocentesis and have intact membranes. The only information on the women available to the investigators was age, race/ethnicity, and number of fetuses present at time of sampling. All clinical assays related to chromosomal assessment of the fetus had been completed, and the residual amniotic fluid and amniotic cell culture pellets were retrieved from the cytogenetics laboratory.

Amniotic fluid samples (8–12 ml) were spun at 4500 rpm for 30 minutes. After decanting the supernatant fluid, the pellet was suspended in 150 μL of TrisEDTA buffer. The cultured cell pellets were resuspended in 200 μL PBS. A 150 μL volume of each sample was extracted using the Roche MagNA Pure LC instrument and the DNA isolation kit I resulting in a final volume of 150 μL.

We used a direct PCR method followed by a nested PCR method to detect HPV. The Roche line blot assay, based on L1 consensus PCR with biotinylated PGMY09/11 primer sets and β-globin as an internal control for sample amplification [17,18] was used as previously described, with 10 μL extract in each 50 μL reaction. All samples were HPV gel-band-negative and Roche Prototype Strip Assay-negative after 40 cycles (reagents provided as a gift from Roche Molecular Systems, Inc., Pleasanton, CA).

For the nested reaction, five microliters of each L1 amplicon was added to a PCR reaction mix containing GP5+/GP6+ [19] primers (1 μM each) and run for an additional 40 cycles under the following conditions: 40 cycles of 94°C for 45 s, 48°C for 4 s, 38°C for 30 s, 42°C for 5 s, 66°C for 5 s, and 71°C for 1.5 min. This was followed by a final extension of 10 min at 72°C. Fifteen μL from each sample was analyzed on a 2% agarose gel.

The HPV assays were repeated using separate extractions, increased volumes of extracts in the PCR assays and re-amplification of L1 consensus PCR products with PGMY primers.

The data was analyzed using SPSS 11 with frequency distributions of study participants by age, race, and ethnicity by HPV status.

## Results

The residual amniotic fluid and cell pellets were collected from 142 women. There were 146 paired samples representing 138 singleton pregnancies, 2 twin pregnancies, 1 triplet pregnancy, and 1 singleton pregnancy with an egg donor. The women's average age was 35.2 years with a range from 23–55 years. The race and ethnicity was 78% Caucasian, 5% African American, 14% Asian, and 2% Hispanic. No other data were available on the women. No HPV was detected in any of the amniotic fluid or pellet cell samples. All samples were β-globin positive. There was no evidence of technical barriers to detecting HPV in samples if it was present.

## Discussion

In this study, no HPV was detected in amniotic fluid or culture cell pellets from 142 pregnant women with intact amniotic membranes. Other investigators have reported the upper 95% confidence interval for detection of perinatal transmission from women with any evidence of genital HPV is only 2.8% [7]. Worda and colleagues found no HPV in amniotic fluid taken just prior to a cesarean delivery in 153 Austrian women with intact membranes [10]. With 142 amniotic samples, we have 80–85% power to detect prevalence of HPV from 1% to 10%. Therefore, the lack of detecting HPV in the amniotic samples is not due to inadequate number of samples.

This study was not able to actually determine the HPV status of the study participants or their risk for having HPV. It is possible that none of the women had ever been infected or were not currently infected with HPV. Other studies addressing the issue of vertical transmission of HPV have reported that 12–36% of pregnant women asymptomatic for HPV were positive for HPV [8,10,16,20]. The population characteristics of these studies were similar to this study with a reported age range of 18–45 years and primarily Caucasian women except studies conducted in Asian countries [8,10,16]. The similarities between our study population and others suggest that 12–36% of the pregnant women should have been infected with HPV [6,8,10,11,16,20,21]. Yet, no HPV was detected in the amniotic fluid.

## Conclusion

Perinatal transmission of HPV clearly does occur; juvenile onset recurrent respiratory papillomatosis is a documented rare outcome of infection with HPV. This occurs in 7 of every 1000 infants born to a mother with genital warts [22]. Some investigators have suggested that infants are exposed and infected by HPV during a vaginal delivery [9]. Studies have documented that there is poor concordance between parents' genital HPV and newborns' oral/genital HPV [20]. In contrast, some investigators have suggested that vertical transmission is possible without evidence of vaginal or cervical secretion contact of the fetus [11-15]. However, this study and others suggest that vertical transmission without exposure to vaginal or cervical secretions is extremely unlikely [8,10].

## Competing interests

The author(s) declare that they have no competing interests.

## Authors' contributions

MR and JB participated in concept development, study design, implementation, and coordination; data analysis, and drafting the manuscript. DR collected the data and coordinated the sample numbers with demographic data. DRL, RAT, DCS, and ERU participated in the HPV assays and drafting the manuscript.

## Pre-publication history

The pre-publication history for this paper can be accessed here:


